# Horizontal Transmission of COVID-19 in a 24-Week Premature Infant and Post-discharge Follow-Up

**DOI:** 10.7759/cureus.18455

**Published:** 2021-10-03

**Authors:** Sweta Bhargava, Rishi Lumba, Pradeep Mally, Sean Bailey, Sourabh Verma

**Affiliations:** 1 Department of Pediatrics, Division of Neonatology, New York University Grossman School of Medicine, New York, USA; 2 Department of Pediatrics, Division of Neonatology, Bellevue Hospital Center, New York, USA

**Keywords:** neonatal intensive care unit, covid-19, premature chronic lung disease, horizontal transmission, extremely premature infants, sars-cov-2

## Abstract

The novel severe acute respiratory syndrome coronavirus 2 (SARS-CoV-2) pandemic has rapidly spread across the globe. The clinical spectrum of infection with SARS-CoV-2 among the most vulnerable extremely premature patient population in the neonatal intensive care units (NICUs), particularly those with chronic lung disease (CLD), remains unclear. Additionally, post-COVID conditions have been described in children with limited published data among infants. Symptoms in children appear similar to those described in the adults. We report a case of SARS-CoV-2 infection in a 24-week preterm infant with CLD acquired via horizontal transmission while still in the NICU. We also provide follow-up data on patient until one year post-discharge. Our patient developed fever prompting testing for SARS-CoV-2. Although extremely premature infants with CLD are known to be at high risk for morbidities if they acquire respiratory viral infections, infection with SARS-CoV-2 in this case report presented with relatively mild clinical symptoms. He remained clinically stable on respiratory support (nasal cannula) with eventual weaning to room air. Our patient was followed until one year post-discharge (chronological age: 20 months) and had follow-up by various subspecialties for chronic lung disease, hypothyroidism, chronic kidney disease, and poor growth. We did not observe any specific post-COVID symptoms. This case illustrates that horizontal transmission of SARS-CoV-2 infection among extremely premature infants with CLD is possible in the NICU but likely presents with mild clinical symptoms during acute infection and less chances of post-COVID conditions. Additionally, this case highlights the need for adherence to infection prevention guidelines to prevent nosocomial transmission amid the ongoing pandemic.

## Introduction

The novel severe acute respiratory syndrome coronavirus 2 (SARS-CoV-2) pandemic causing coronavirus disease 2019 (COVID-19) has rapidly spread across the globe. Infants and children are as likely as adults to get infected but have fewer symptoms and less severe clinical course [1,2]. The possibility of vertical transmission from pregnant women with SARS-CoV-2 to their newborns appears less likely [3]. More literature is needed to understand the possibility of vertical transmission [4,5]. Few published case reports of confirmed SARS-CoV-2 infection among neonates born to mothers with active SARS-CoV-2 infection have generally shown an asymptomatic or mild clinical course with a rare need for mechanical ventilation [6,7]. The clinical spectrum of infection with SARS-CoV-2 among the most vulnerable extremely premature patient population who are still admitted to the neonatal intensive care units (NICUs), particularly those with chronic lung disease (CLD), remains unknown at this time. Post-COVID conditions have been described in adults with combinations of health problems similar to post-acute viral syndromes previously noted in past virulent coronavirus epidemics [8]. In children, there are limited data showing possible post-COVID conditions. Symptomatology of post-COVID conditions among children seems similar to those described in adults (fatigue, muscle and joint pain, headache, insomnia, respiratory problems, and palpitations), although larger prospective studies are needed to further study long-term effects of COVID-19 in children [9]. These symptoms may be even more difficult to assess in high-risk neonatal population who have had prolonged hospitalizations. We describe here a case of SARS-CoV-2 infection in a 24-week preterm infant with chronic lung disease acquired via horizontal transmission while still in the NICU and subsequent one-year follow-up after discharge until 20 months of chronological age.

## Case presentation

A male infant was born by cesarean section at 24 weeks and 5 days gestational age, with a birth weight of 700 grams, to a 29-year-old mother (gravida 4, parity 1, preterm 1, miscarriage 1, liveborn 1) with history of pituitary microadenoma and hyperprolactinemia. Mother presented with advanced cervical dilation and footling breech presentation leading to emergency cesarean section. Apgar scores were 4 and 7 at one and five minutes of life, respectively. He was intubated in the delivery room, and he required mechanical ventilation and surfactant for respiratory distress syndrome of prematurity in the NICU. The patient was transferred to our facility at one week of age for escalation of care due to spontaneous intestinal perforation.

He had a complicated respiratory course during hospitalization including an episode of pulmonary hemorrhage, prolonged mechanical ventilation, and development of CLD of prematurity. He was finally extubated successfully by 10 weeks of age and gradually weaned off respiratory support to nasal cannula by five months of age. He had a hemodynamically significant patent ductus arteriosus, which required surgical ligation after failed medical management. The patient had spontaneous intestinal perforation at one week of age complicated by fungal peritonitis and complex ascites requiring primary peritoneal drainage, antifungal treatment, and eventual exploratory laparotomy. This resulted in delayed feeding initiation by two months of age. All head sonograms were normal for age. He had progressive retinopathy of prematurity (ROP) for which he received bevacizumab (Avastin®, Genetech, Inc., San Francisco, California, USA) followed by laser photocoagulation. At five months of chronological age, he continued to require respiratory support with nasal cannula (flow: 2 Liters/min; fraction of inspired oxygen: 0.30). He received budesonide and diuretics for the management of chronic lung disease. He was tolerating full enteral feeds of low mineral formula Similac® PM 60/40 (Abbott Nutrition, Columbus, Ohio, USA; for abnormal electrolytes due to multifactorial chronic renal disease), getting vitamin D and calcium supplementation for osteopenia of prematurity, and getting levothyroxine for hypothyroxinemia of prematurity.

**Figure 1:**
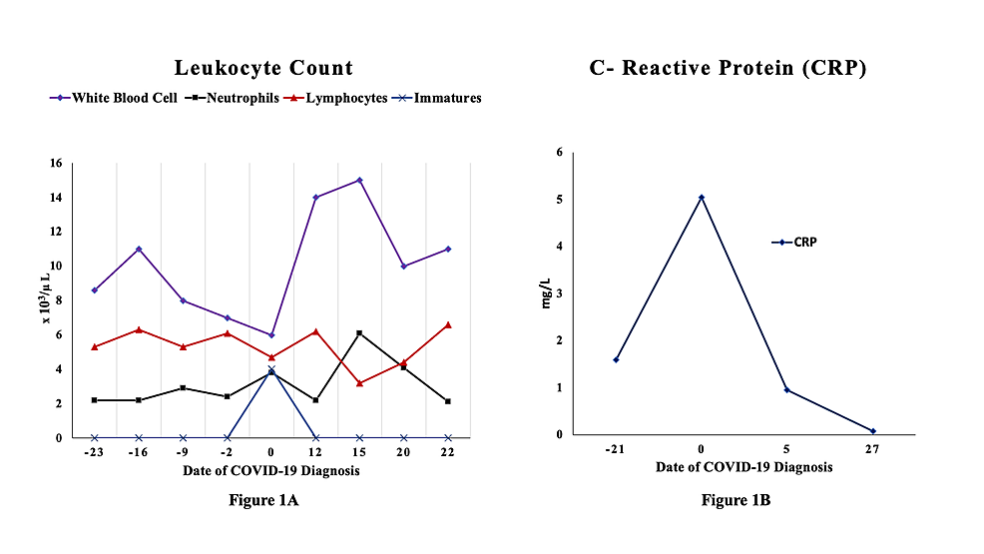
Leukocyte count and C-reactive protein levels Leukocyte count (A) and C-reactive protein levels (B). CRP: reference range: <5 mg/L. CRP: C-reactive protein, COVID-19: coronavirus disease 2019.

**Figure 2:**
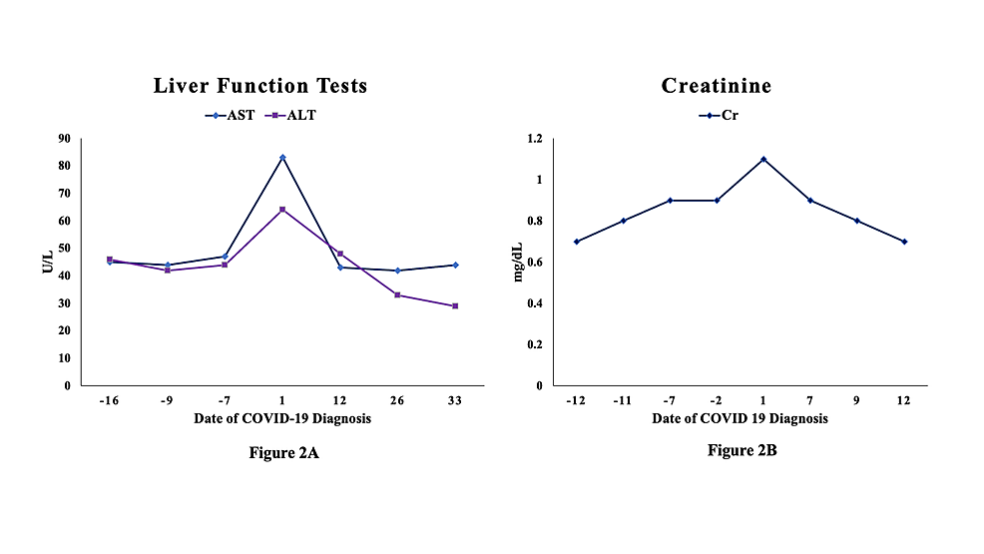
Liver and kidney function tests Liver (A) and kidney function (B) tests. AST: reference: <50 U/L; ALT: reference: <50 U/L; Cr: reference: 0.5-0.9 mg/dL. AST: aspartate transaminase, ALT: alanine transaminase, Cr: creatinine, COVID-19: coronavirus disease 2019.

**Figure 3:**
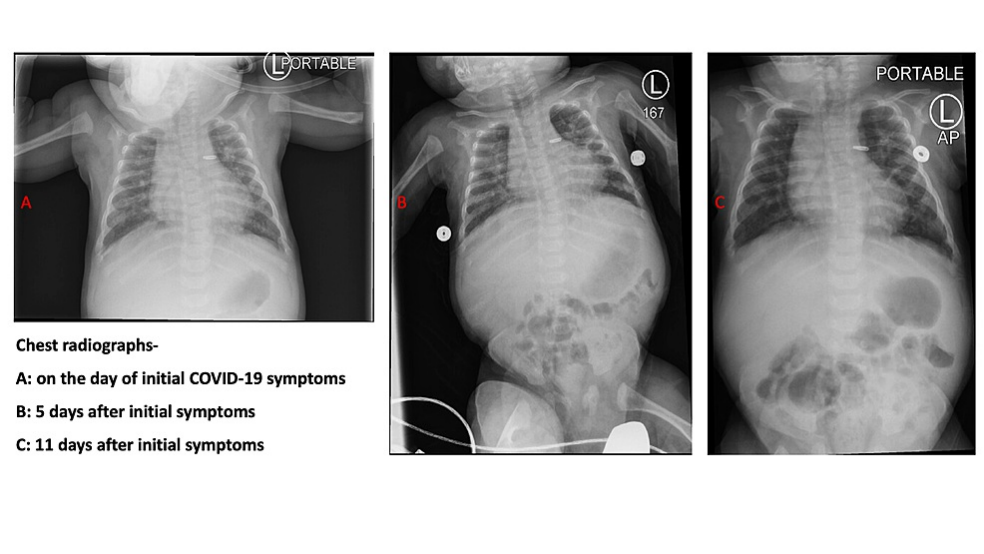
Serial chest radiographs showing the progression of pulmonary disease after diagnosis of COVID-19 Perihilar interstitial infiltrates with subsegmental atelectasis (A and B), which can be seen in viral infections. These findings were resolved over time (C). (A) On the day of initial COVID-19 symptoms, (B) five days after initial symptoms, and (C) 11 days after initial symptoms. Patent ductus arteriosus ligation clip in situ on the radiograph. COVID-19: coronavirus disease 2019, AP: anteroposterior view.

At chronological age of five months, he developed fever (temperature 38.7° Celsius) prompting a partial sepsis workup including blood and urine culture with initiation of empiric broad-spectrum antibiotics. A respiratory viral panel (using BioFire® FilmArray® respiratory panel, bioMérieux, Salt Lake City, Utah, USA) and a real-time reverse transcription polymerase chain reaction of nasopharyngeal swab for SARS-CoV-2 (BioReference Laboratories, Elmwood Park, New Jersey, USA) were sent in view of widespread community SARS-CoV-2 transmission and history of sick contact with mother and sibling. Both were symptomatic with mild cough and fever in the preceding days but were not tested for SARS-CoV-2. Additionally, the patient’s mother had recently visited the NICU but reported no symptoms at the time of visit. Physical examination of the infant showed mild intermittent tachypnea with no other significant findings, accompanied by no change in his overall respiratory support or oxygen requirement (flow: 2 Liters/min; fraction of inspired oxygen: 0.30). He continued to tolerate feeds without any feeding intolerance. Initial laboratory results were notable for mild leukopenia (white blood cells count: 6 x10^3^/µL) and anemia of prematurity (hemoglobin: 8.8 g/dL). Absolute lymphocyte counts (3,008 cells/ µL, reference: <1,500 cells/ µL) and C-reactive protein (CRP, 5.1 mg/L, reference: <5 mg/L) were normal at the time of partial sepsis workup. Additionally, mild elevation of liver enzymes and creatinine was noted at the time of diagnosis (Figures 1A, 1B, 2A, 2B). A chest radiograph showed increasing perihilar airspace opacities that could represent underlying viral or atypical pneumonitis (Figure 3A-3C).

 

He was placed in a negative pressure isolation room with contact, airborne, and eye protection (goggles or face shield) precautions. Patient’s nasopharyngeal swab was positive for SARS-CoV-2 the same day. Respiratory viral panel was negative for other common respiratory viruses. The blood and urine cultures were resulted negative for any growth.

**Figure 4:**
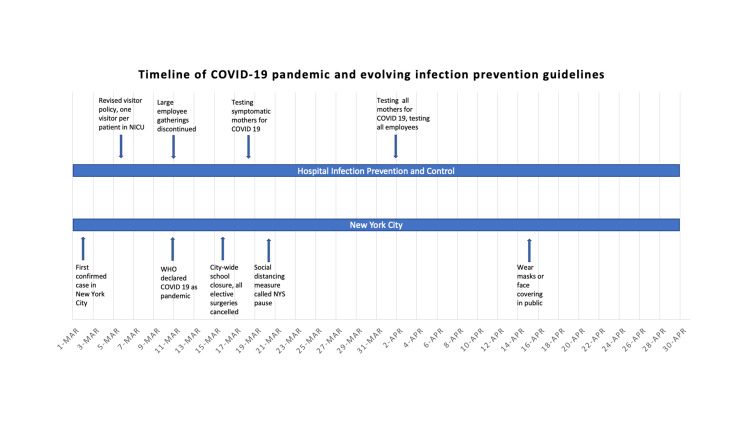
Timeline of COVID-19 pandemic in year 2020 and evolving hospital and local health departmental infection prevention guidelines COVID-19: coronavirus disease 2019, MAR: March, APR: April, NYS: New York State, NICU: neonatal intensive care unit.

Initial blood gas analysis at the time of testing for SARS-CoV-2 was within normal limits. Subsequent blood gas analyses showed a trend toward mild metabolic acidosis with respiratory compensation, normal capillary pH, low bicarbonate level of 17.8 mmol/L (reference: 25 to 32 mmol/L), and base excess of negative 6.8 mmol/L (reference: +2.0 to -2.0 mmol/L) on day 5 after testing for SARS-CoV-2, which improved on the following days. The longitudinal measurements of laboratory indices over next few days such as CRP, complete blood cells count, and renal and hepatic function tests remained normal for age (Figures 1A, 1B, 2A, 2B). Pediatric infectious disease team was consulted regarding management of this patient. Considering his overall stable cardiorespiratory status, no targeted experimental treatment for COVID-19 was initiated. The repeat second and third tests of nasopharyngeal swab for SARS-CoV-2 real-time reverse transcription polymerase chain reaction (RT-PCR) were positive (days 5 and 10 after initial testing) and finally turned negative on the fourth test (day 15 after initial testing). There had been no cases of SARS-CoV-2 infection in the NICU before and after the index case. Following the diagnosis of COVID-19 in this patient, the parents were temporarily restricted from visiting the unit as per our local infection prevention guidelines [10]. Notably, one healthcare worker developed upper respiratory symptoms a few days after this infant’s diagnosis and tested positive for SARS-CoV-2. A timeline of COVID-19 diagnosis in the beginning of pandemic and evolving infection prevention guidelines within our hospital system and New York City health department is shown in Figure 4. At discharge from the NICU, he was stable on room air and taking full feeds by mouth of Similac® PM 60/40 (24 calories per fluid ounce) due to his history of chronic kidney disease. He was on Synthroid for his hypothyroidism, vitamin D for osteopenia of prematurity, and amoxicillin for his history of urinary tract infection and chronic renal disease. All head ultrasounds obtained throughout his admission were normal.

Since his discharge, he had various sub-specialists follow-up visits. He was seen by pediatric pulmonology for his CLD and remains stable on room air. He is followed by pediatric nephrology for chronic renal disease, and his feeds were changed gradually to Enfamil® Neuro Pro™ (Mead Johnson & Company, LLC; Chicago, Illinois, USA) from Similac® PM 60/40. He has been off amoxicillin prophylaxis by 11 months of chronological age. He is followed by pediatric endocrinology for his hypothyroidism and was on Synthroid until 13 months of age, at which time his medication was discontinued with recent thyroid function tests resulting normal for age. He continues to be followed by ophthalmology and had regressed ROP on his last visit at one year of chronological age. By chronological age of 20 months, he can crawl and stand with support, which he started at 18 months of age. He speaks only a few words such as dada and mama. He is getting physical therapy for 30 minutes two times a week via the ‘Early Intervention’ program but is referred for assessing need for occupational or speech therapy. He has had issues with feeding intolerance, with spit up/emesis with feeds because of which he has poor weight gain and growth. He is being followed by nutrition services to help with optimization of growth. He is now eating solid food well with formula supplementation. He is up to date on his vaccines. He has not had any cough, shortness of breath or hospitalizations since his discharge from the NICU. Of note, he has had multiple emergency department (ED) visits for increased irritability, emesis, and subjective fever at home. During those visits, he had no respiratory distress, rash, no vital sign abnormalities in the ED, and no fever documented in ED. He was tested for COVID-19 on those visits with all results being negative.

## Discussion

We report a case of COVID-19 in a 24-week premature neonate with CLD via horizontal transmission, while he was still admitted to the NICU. Additionally, we provide one-year post-discharge follow-up report and did not observe any specific post-COVID symptoms in this patient. Although extremely premature infants with chronic lung disease are known to be at high risk for morbidities if they acquire respiratory viral infections during their NICU stay, infection with SARS-CoV-2 in this case report presented with relatively mild clinical symptoms. Nevertheless, this case shows that horizontal transmission of SARS-CoV-2 infection is possible in the NICU setting, especially in the areas with high transmission rates. Therefore, proactive infection prevention and control guidelines are warranted in all NICUs in the currently ongoing pandemic.

There is a paucity of data describing clinical spectrum among neonates with confirmed SARS-CoV-2. The focus to date has been to evaluate the possibility of vertical transmission from mothers to their newborns [3]. A few case reports and small case series describing SARS-CoV-2 infection among neonates via horizontal transmission had infants who were already discharged from the hospital and potentially got infected after being in touch with the other households [6,7]. In a systematic review of neonatal cases with SARS-CoV-2, most of these patients were asymptomatic or had mild to moderate signs of clinical infection [1]. Rarely, newborns with SARS-CoV-2 have been reported to require mechanical ventilation and aggressive therapies as well [1]. A case of SARS-CoV-2 infection via horizontal transmission was recently reported in a 27-week premature infant at eight weeks of age, who was at home after discharge from the NICU [6]. This infant presented to the emergency department with poor feeding, sneezing, and dyspnea. The patient had further clinical deterioration requiring intubation and high-frequency ventilation for respiratory failure and inhaled nitric oxide for pulmonary hypertension. But in this case, patient also had concomitant positive blood culture that may have been the primary reason for the level of severity of illness. Another case of a 26-week premature infant has been reported; in this case, the infant’s mother was diagnosed with COVID-19 on day 7 after delivery. The mother had skin-to-skin contact with the infant prior to diagnosis. This infant remained stable with no temperature instability or increase in baseline respiratory requirements [7].

The patient in our case report developed relatively milder symptoms consistent with these reports, despite having multiple comorbidities such as prematurity, chronic lung disease, and inadequate anthropometric growth. Radiographic findings of perihilar opacities described in our patient (Figure 1) have been described among young children with COVID-19 [11]. Inflammatory markers such as CRP and white blood cell counts were not significantly increased in our case (Figure 2), which can be abnormal in children with severe infection. We observed mild metabolic acidosis in the index case by day 5 of initial testing, which could be seen with viral infections. Patient’s overall cardiorespiratory status stayed stable after infection with SARS-CoV-2. Infants have been observed to have higher nasopharyngeal viral load compared to older children, but the rate of contracting severe illness is lesser than the older children and adults with SARS-CoV-2 [12]. The exact causes for these differential outcomes are yet to be determined. Some possible explanations could be due to evolving immunological response to the viral infections in early life and age-dependent differential expression of cell surface angiotensin-converting enzyme 2 receptors, which is important for the entry of SARS-CoV-2 virus into cells [13]. Although it is difficult to ascertain if increased irritability and feeding intolerance noted in this patient is a result of or long-term effects of COVID-19, it seems more likely due to his history of necrotizing enterocolitis and microcolon. Based on current follow-up, it is unlikely that our patient has had any specific post-COVID conditions.

The patient described in this report acquired SARS-CoV-2 while still admitted to the NICU and would be considered a nosocomial infection. At the start of pandemic spread in the United States by early March, there was inadequate guidance available regarding infection prevention control in the NICU setting. The testing for SARS-CoV-2 was extremely limited nationwide and was based on symptoms screening as defined by the Centers for Disease Control and Prevention. Subsequently, more testing became available due to Emergency Use Authorization by The Food and Drug Administration. We developed initial guidance documents that continued to evolve as more information became available regarding SARS-CoV-2 infection, including guidance from the American Academy of Pediatrics [10,14]. In view of widespread community transmission, there is a concern for unintended exposure from family and healthcare workers, especially to the vulnerable patient population in the NICU. At our institutions, we decided to test for SARS-CoV-2 in infants whenever there was a concern for a respiratory viral infection with no alternate explanation or diagnosis. In addition, the visitation guidelines were changed to maximum of two individuals with only one person at a time at the bedside (either of the parents or two assigned individuals per family). Parents and staff were screened every day for temperature, symptoms, and travel history prior to entry to the hospital. Wearing face masks was made compulsory for all healthcare workers and family members at all times. Strict infection prevention guidelines were put in place while caring for all patients with frequent auditing for compliance. Infection prevention and control and pediatric infectious disease teams are consulted for further patient-specific guidance on repeat testing and maintenance or discontinuation of isolation precautions.

## Conclusions

We report a case of COVID-19 in a 24-week extremely premature neonate with chronic lung disease who acquired infection via horizontal transmission while still admitted to the NICU. Although extremely premature infants with chronic lung disease are at high risk for morbidities if they acquire respiratory viral infections, SARS-CoV-2 infection presented with mild clinical symptoms in the index case of this report. Although no significant post-COVID conditions were noted in our patient, further studies are needed to understand long-term impact of SARS-CoV-2 infection in children. This case highlights that horizontal transmission of SARS-CoV-2 infection is possible in the NICU setting, especially in the areas with high transmission rates; therefore, proactive infection prevention and control guidelines are warranted in all NICUs in currently ongoing pandemic.
